# Web- and Artificial Intelligence–Based Image Recognition For Sperm Motility Analysis: Verification Study

**DOI:** 10.2196/20031

**Published:** 2020-11-19

**Authors:** Vincent FS Tsai, Bin Zhuang, Yuan-Hung Pong, Ju-Ton Hsieh, Hong-Chiang Chang

**Affiliations:** 1 Department of Urology Ten-Chan General Hospital Taoyuan Taiwan; 2 Department of Urology National Taiwan University Hospital Taipei Taiwan; 3 Division of Research and Development Createcare Technology Corporation Shenzhen China; 4 Department of Urology Ten-Chen General Hospital Taoyuan Taiwan

**Keywords:** Male infertility, semen analysis, home sperm test, smartphone, artificial intelligence, cloud computing, telemedicine

## Abstract

**Background:**

Human sperm quality fluctuates over time. Therefore, it is crucial for couples preparing for natural pregnancy to monitor sperm motility.

**Objective:**

This study verified the performance of an artificial intelligence–based image recognition and cloud computing sperm motility testing system (Bemaner, Createcare) composed of microscope and microfluidic modules and designed to adapt to different types of smartphones.

**Methods:**

Sperm videos were captured and uploaded to the cloud with an app. Analysis of sperm motility was performed by an artificial intelligence–based image recognition algorithm then results were displayed. According to the number of motile sperm in the vision field, 47 (deidentified) videos of sperm were scored using 6 grades (0-5) by a male-fertility expert with 10 years of experience. Pearson product-moment correlation was calculated between the grades and the results (concentration of total sperm, concentration of motile sperm, and motility percentage) computed by the system.

**Results:**

Good correlation was demonstrated between the grades and results computed by the system for concentration of total sperm (r=0.65, *P*<.001), concentration of motile sperm (r=0.84, *P*<.001), and motility percentage (r=0.90, *P*<.001).

**Conclusions:**

This smartphone-based sperm motility test (Bemaner) accurately measures motility-related parameters and could potentially be applied toward the following fields: male infertility detection, sperm quality test during preparation for pregnancy, and infertility treatment monitoring. With frequent at-home testing, more data can be collected to help make clinical decisions and to conduct epidemiological research.

## Introduction

Infertility is a worldwide problem, with a prevalence of 15% [1], and sperm plays an important role [2]. In the process of fertilization, sperm are initially ejaculated around the cervix and then swim to the proximal oviduct, where they encounter the oocyte and accomplish fertilization [3]. To accomplish fertilization, large numbers of sperm are needed to overcome the filtration of cervical mucus while progressive motility is necessary for sperm to travel for a long distance. Human sperm concentration and motility fluctuate over time [4]. Therefore, frequent monitoring of sperm concentration and motility is crucial for couples who are preparing for spontaneous pregnancy.

Conventionally, men are asked to have semen analysis performed at a doctor's office or laboratory, a procedure that is both time-consuming and embarrassing for men, according to our clinical observations and prior literature [5]. Hence, it makes frequent measurement of sperm concentration and motility difficult. To tackle this problem, several trials of home sperm tests were developed in the past few decades, such as SpermCheck [6], Fertell [7], and Trak Male Fertility Testing System [8]. Some, such as YO sperm test and the Kobori single ball-lens system, attracted a lot of attention; both are systems that utilizes platforms based on smartphone systems [9,10]. There are some advantages to using smartphone-based home sperm tests, such as high-resolution video recording, robust calculation ability, and accessible internet communication. All these advantages can allow users to be tested in a setting that encourages more frequent measurements, while preserving the privacy and accuracy of results, similar to those of other point-of-care test systems that have been applied in measurements of blood sugar, blood pressure, and body temperature.

**Figure 1:**
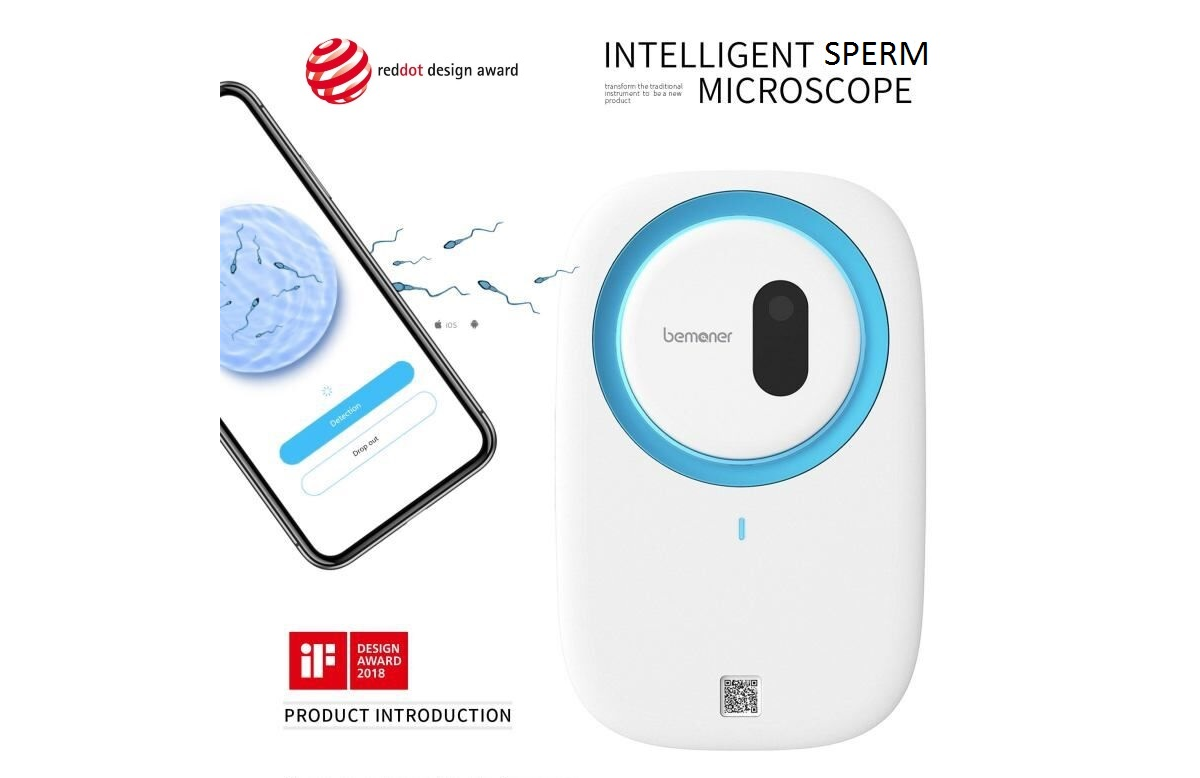
Bemaner smartphone-based home sperm motility test system.

Bemaner (Shenzhen Createcare Technology Co) is a smartphone-based home sperm motility measurement system composed of a microscope and microfluidic modules and is designed to adapt to different types of smartphone designs and interfaces (Figure 1). Bemaner is quite different from the YO sperm test and the Kobori single ball-lens system [10]. The YO sperm test utilizes a tailor-made adapter (slide) to fit specific types of smartphones, but Bemaner can fit all smartphones currently. Sperm videos are captured and uploaded to the cloud from the smartphone via software apps. Unlike the Kobori single ball-lens system, in which motile and static sperm are counted by a person [10], for Bemaner, analysis of sperm motility is performed by an artificial intelligence (AI) image recognition algorithm, and results are displayed to end users on the smartphone interface. The purpose of this study was to verify the performance of the AI sperm image recognition algorithm and cloud computing system.

## Methods

### Video Clips of Motile Sperm and Results Computed by AI

Semen samples can be collected at home by users. After 30 minutes of liquefaction, the semen sample is dipped by a small biochip cup (analog to glass slide) and then covered by a large biochip cup (analog to cover slip). The space containing semen samples between these two cups is designed to be 10 micrometers deep, which can contain a single layer of sperm for a specific volume of 0.2 microliters. Through the microscopic and microfluidic modules of the device, the video clips of motile sperm can be captured and uploaded by any recent smartphone, which is easily aligned to the microscopic and microfluidic modules (Multimedia Appendix 1). Deidentified (ie, users' information removed) video clips of motile sperm were retrieved from the central cloud computing server (Alibaba cloud, Hangzhou, China). With the process of deidentification, it was impossible to obtain specific user consent for this study. However, the security and privacy of users' information were well protected through the process of deidentification.

The results, including the concentration of total sperm, the concentration of motile sperm, and the motility percentage, computed by the AI image recognition algorithm (version 1.0.8_5/22) with cloud computing on the central server were also retrieved with every video clip of motile sperm. In detail, concentration of total sperm and motile sperm were derived by the image recognition AI algorithm. Motility percentage is calculated as the quotient of concentration of motile to total sperm.

### Scoring of Motile Sperm

The gold standard for assessing semen quality in the current World Health Organization manual [4] is analysis performed under a microscope by a well-trained professional staff. According to the number of motile sperm in the vision field of each video clip, the 47 deidentified videos of motile sperm were classified into 6 grades (0-5) by a male-fertility expert with 10 years of experience. The criteria were the following: grade 0, there are no areas of motile sperm in the field of vision; grade 1, some motile sperm; grade 2; the amount of motile sperm is between grade 1 and 3; grade 3, motile sperm occupy half of the field of vision; grade 4, the number of motile sperm is between grade 3 and 5; grade 5, motile sperm occupy almost the whole field of vision (Multimedia Appendix 2-Multimedia Appendix 7).

### Relationship Between Human and AI Results

The grade assessed by a male-fertility expert was an ordinal variable; the relationships between grade and concentration of total sperm, concentration of motile sperm, and motility percentage was calculated determined with Pearson product-moment correlation coefficients. Analyses were calculated using the software Excel (Microsoft Inc, 2013). A *P* value less than .01 was considered statistically significant.

## Results

The results (concentration of total sperm, concentration of motile sperm and motility percentage) of the AI algorithm and the distribution of scored grade according to the number of motile sperm in the vision field of each video clip are is shown in Table 1.

**Figure 2:**
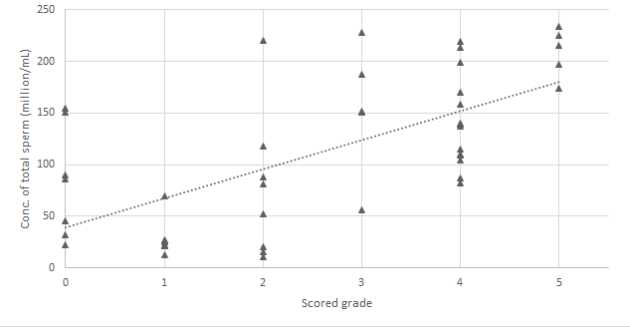
Relationship between Bemaner concentration of total sperm and scored grades.

**Figure 3:**
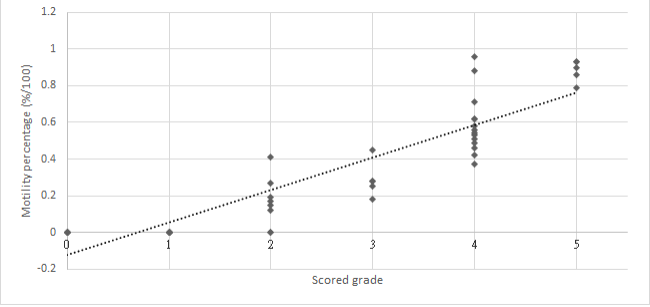
Relationships between Bemaner motility percentage and scored grades.

**Figure 4:**
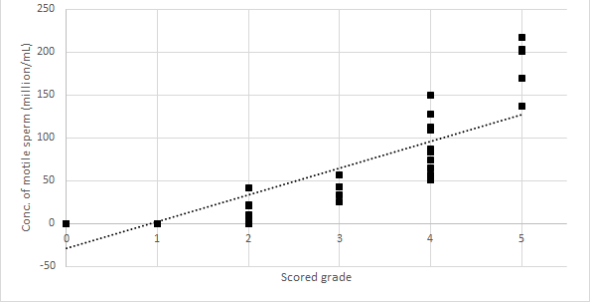
Relationships between Bemaner concentration of motile sperm and scored grades.

Relationships between the grades and Bemaner AI algorithm results were *r*=0.65 (*P*<.001) for concentration of total sperm (Figure 2), *r*=0.90 (*P*<.001) for motility percentage (Figure 3), and *r*=0.84 (*P*<.001) for concentration of motile sperm (Figure 4).

**Table 1:** Results.

Source and variable		Value
**Bemaner AI algorithm-based, mean (SD)**		
	Concentration of total sperm (million cell/mL)	111.02 (72.13)
	Concentration of motile sperm (million cell/mL)	50.87 (61.47)
	Motility percentage (%)	32.8 (32.5)
**Sperm motility count by expert grade (million cell/mL), n**		
	Grade 0	7
	Grade 1	8
	Grade 2	8
	Grade 3	5
	Grade 4	14
	Grade 5	5

## Discussion

To our knowledge, this is the first smartphone-based system for semen analysis accomplished through an AI image recognition algorithm on cloud computing. Even though there are other smartphone-based systems, such as YO sperm test [9] and Kobori single ball-lens system [10], neither utilizes an AI imaging recognition algorithm with cloud computing. YO sperm test analyzes sperm videos by an app installed on a smartphone, while Kobori single ball-lens system projects the mobile phone captured videos onto a desktop monitor and counts sperm parameters manually.

Additionally, there are some benefits for data processing through cloud computing. Only requiring connection with the internet, the system provides an increased flexibility and possibility in terms of setting up test locations and test frequencies. It is also easier for engineers to perform system maintenance such as algorithm revision and software updates instead of frequent update requests on the user end smartphone app. Also, accumulated big data may demonstrate important epidemiological characteristics of male fertility (eg, regional or temporal). Such data processes, based on cloud computing, are quite novel and promising for large-scaled epidemiological studies and public-health related policy making.

The results calculated by Bemaner and grades assessed by a male-fertility expert, motility percentage and concentration of motile sperm, demonstrated better correlations with the scored grade. The respective correlation coefficients are 0.90 (*P*<.001) and 0.84 (*P*<.001). This means that motility percentage and concentration of motile sperm calculated by Bemaner were comparable to a male-fertility expert's judgment for assessing sperm motility.

However, concentration of total sperm was only moderately correlated (*r*=0.65; *P*<.001) with the scored grade. This is attributed to some of total sperm being immobile sperm, which cannot achieve natural fertilization [11,12].

With regard to corresponding motile sperm concentration of each scored grade, the calibrated reference values of sperm concentration are provided (Table 2).

**Table 2:** Calibrated reference values of sperm concentration.

Grade	Range (million cells/mL)
0	0
1	<13.9
2	0-27.7
3	28.3-51.9
4	50.7-113.9
5	153.4-217.6

The accuracy of these values can be improved as the accumulated data size grows. This is also one of the benefits that these types of online processes for point-of-care test results can provide.

In comparison with other smartphone-based sperm analysis systems, such as YO sperm test [9] and the Kobori single ball-lens system [10], Bemaner can provide users with information about sperm concentration, motile sperm percentage, and motile sperm concentration. These parameters are essential for a man to achieve natural fertilization. That may be the reason why these parameters are chosen for screening tests [13,14].

Graded results show more information about fluctuation of sperm concentration and motility than results indicating positive or negative, such as SpermCheck [6] and Fertell [7]. It is also more easily understood by lay users. YO sperm test and Bemaner, in the latest version of app on the smartphone provides not only total sperm concentration, motile sperm concentration, and percentage of motile sperm but also graded results of the motile sperm concentration based on the findings of this study. Both provide graded results for motile sperm concentration (ie, YO score and Bemaner scored grade). It can be used as a home sperm test instead of having comprehensive and robust sperm parameters assessments in a fertility laboratory [4]. Practically, graded results can be applied in some scenarios that require information about fluctuating test results, such as male infertility detection, sperm quality test during preparation for pregnancy, and infertility treatment monitoring.

YO sperm test obtained test results by the installed algorithm and the results were compared to those of another image-recognition sperm test SQA-Vision (Medical Electronics Systems) by Agarwal et al [9]. Kobori et al [10] counted sperm on images projected to desktop screen and compared their results with computer assisted semen analysis. In contrast, Bemaner used an AI algorithm with cloud computing to analyze images for sperm testing. The results were compared with the grades assessed by an experienced male-fertility expert. Because the images for analysis were uploaded by end users and deidentified, it was impossible to acquire any original parameters of the corresponding semen samples. (The appearance, viscosity, pH, total sperm concentration and motile sperm concentration manually measured under microscope for a semen sample are original parameters of the sample. Only the previous captured and uploaded videos of sperm could be retrieved in this study.) Therefore, grades of sperm images were utilized as the reference method. This is one of the limitations of the study.

Currently, the aforementioned 3 systems cannot analyze sperm images to assess sperm morphology according to the World Health Organization morphology assessment paradigm [4]. A new version of the AI algorithm including morphology assessment is currently in development for the Bemaner system.

In the face of emerging pandemics causing city lockdown and health care need for remote regions, tele-medicine will prevail and requires on-line processes of data and analysis connected with off-line point-of-care tests for end users. The Bemaner system is a prime example of this type of implementation, especially for infertility, which is always related to the following aspects: environmental pollution, life style, and diet [15-17], the analysis of big data could contribute much more than traditional case reports in research [18].

The framework of data processing in this system can be reconstructed as the big data grows and we extend our scope to collect the different aspects related to users' life style, diet, environmental pollutions, and medical interventions. In the future, some techniques of cloud computing and data storage to optimize big data processing could be applied to help speed the process, such as adopting concepts such as intermediate data caching. Nevertheless, the current process of data transmission and analyzing is similar to an application program interface call, in which a request with the data is sent to the server, the server processes the data with our image recognition algorithm to generate the result, and the server responses with an answer.

There are still some improvements to be implemented in the near future. The biochip cups for containing semen specimens could be adjusted automatically by a step-motor to increase the scope of observation and the number of observed sperm. The optic module could be modified to increase the magnification to define sperm's morphology according to the WHO criteria [4]. Revisions of the AI image recognition algorithm will continue to improve as data accumulates, enhancing its accuracy. Big data for this system has huge potential to introduce various insight, both in clinical and epidemiological fields, to improve human fertility.

This smartphone-based system for measuring sperm motility (Bemaner) accurately measures parameters related to sperm motility and could potentially be applied toward the following fields: male infertility detection, sperm quality test during preparation for pregnancy, and infertility treatment monitoring. With frequent testing, more data can be collected to help make clinical decisions and conduct epidemiological studies.

The overall contributions of this paper are (1) to introduce a novel online system for semen analysis calculated by an AI image recognition algorithm combined with cloud computing technology; (2) to prove the performance of the system by correlating human and AI results; (3) to suggest potential applications in male infertility detection, sperm quality test during preparation for pregnancy, and infertility treatment monitoring; and (4) to foresee that the big data collected by this system can play an important role in making clinical decisions and conducting epidemiological research.

## Appendix

Multimedia Appendix 1 Operating process of Bemaner.

Multimedia Appendix 2 Scored grades of sperm videos: Grade 0.

Multimedia Appendix 3 Scored grades of sperm videos: Grade 1.

Multimedia Appendix 4 Scored grades of sperm videos: Grade 2.

Multimedia Appendix 5 Scored grades of sperm videos: Grade 3.

Multimedia Appendix 6 Scored grades of sperm videos: Grade 4.

Multimedia Appendix 7 Scored grades of sperm videos: Grade 5.

## Abbreviations

AI: artificial intelligence
WHO: World Health Organization

## References

[1] Agarwal A, Mulgund A, Hamada A, Chyatte MR (2015). A unique view on male infertility around the globe. Reprod Biol Endocrinol.

[2] Nosrati R, Graham PJ, Zhang B, Riordon J, Lagunov A, Hannam TG, Escobedo C, Jarvi K, Sinton D (2017). Microfluidics for sperm analysis and selection. Nat Rev Urol.

[3] Suarez S, Pacey A (2006). Sperm transport in the female reproductive tract. Hum Reprod Update.

[4] (2010). Laboratory manual for the examination and processing of human semen. 5th edition.

[5] Elzanaty S, Malm J (2008). Comparison of semen parameters in samples collected by masturbation at a clinic and at home. Fertil Steril.

[6] Coppola M, Klotz K, Kim K, Cho H, Kang J, Shetty J, Howards S S, Flickinger C J, Herr J C (2010). SpermCheck Fertility, an immunodiagnostic home test that detects normozoospermia and severe oligozoospermia. Hum Reprod.

[7] Björndahl L, Kirkman-Brown J, Hart G, Rattle S, Barratt C (2006). Development of a novel home sperm test. Hum Reprod.

[8] Schaff UY, Fredriksen LL, Epperson JG, Quebral TR, Naab S, Sarno MJ, Eisenberg ML, Sommer GJ (2017). Novel centrifugal technology for measuring sperm concentration in the home. Fertil Steril.

[9] Agarwal A, Panner Selvam MK, Sharma R, Master K, Sharma A, Gupta S, Henkel R (2018). Home sperm testing device versus laboratory sperm quality analyzer: comparison of motile sperm concentration. Fertil Steril.

[10] Kobori Y, Pfanner P, Prins GS, Niederberger C (2016). Novel device for male infertility screening with single-ball lens microscope and smartphone. Fertil Steril.

[11] Ombelet W, Dhont N, Thijssen A, Bosmans E, Kruger T (2014). Semen quality and prediction of IUI success in male subfertility: a systematic review. Reprod Biomed Online.

[12] Wang C, Swerdloff RS (2014). Limitations of semen analysis as a test of male fertility and anticipated needs from newer tests. Fertil Steril.

[13] Buck Louis GM, Sundaram R, Schisterman EF, Sweeney A, Lynch CD, Kim S, Maisog JM, Gore-Langton R, Eisenberg ML, Chen Z (2014). Semen quality and time to pregnancy: the Longitudinal Investigation of Fertility and the Environment Study. Fertil Steril.

[14] Almeida S, Rato L, Sousa M, Alves MG, Oliveira PF (2017). Fertility and sperm quality in the aging male. Curr Pharm Des.

[15] Nassan FL, Jensen TK, Priskorn L, Halldorsson TI, Chavarro JE, Jørgensen Niels (2020). Association of dietary patterns with testicular function in young Danish men. JAMA Netw Open.

[16] Kasman AM, Del Giudice F, Eisenberg ML (2020). New insights to guide patient care: the bidirectional relationship between male infertility and male health. Fertil Steril.

[17] Poli D, Andreoli R, Moscato L, Pelà Giovanna, de Palma Giuseppe, Cavallo D, Petyx M, Pelosi G, Corradi M, Goldoni M (2020). The relationship between widespread pollution exposure and oxidized products of nucleic acids in seminal plasma and urine in males attending a fertility center. Int J Environ Res Public Health.

[18] Patel DP, Jenkins TG, Aston KI, Guo J, Pastuszak AW, Hanson HA, Hotaling JM (2020). Harnessing the full potential of reproductive genetics and epigenetics for male infertility in the era of "big data". Fertil Steril.

